# Care partner experience with telepresence robots in long-term care during COVID-19 pandemic

**DOI:** 10.1177/20552076251319820

**Published:** 2025-02-05

**Authors:** Grace Hu, Joey Wong, Lily Haopu Ren, Sarah Kleiss, Annette Berndt, Lily Wong, Ali Hussein, Nazia Ahmed, Jim Mann, Lillian Hung

**Affiliations:** 1 Innovation in Dementia and Aging Lab, 8166 University of British Columbia, Vancouver, BC, Canada; 2 27177 Universitätsmedizin Göttingen, Göttingen, Germany

**Keywords:** Family caregivers, older adults, nursing homes, assistive technology, COVID-19

## Abstract

**Objective:**

As people living with dementia move into long-term care (LTC), their care partners face a difficult role change from primary caregiver to visitor, losing a significant degree of control and direct care involvement. The COVID-19 pandemic exacerbated these challenges with health risks, changing care home protocols, and government policies. To help address these challenges, this study aimed to investigate the experiences of care partners who used telepresence robots to maintain contact with and care for their loved ones during the pandemic.

**Methods:**

This study was guided by the Collaborative Action Research (CAR) approach. Along with interdisciplinary researchers and trainees, our team included patient and family partners as co-researchers throughout the project. We conducted semi-structured interviews with 20 care partners who used the robots in five urban Canadian LTC homes between May 2021 and August 2023.

**Results:**

Thematic analysis identified four key themes characterizing their experiences using the robot: (a) decreases care partner burden, (b) facilitates care partner–staff relationship, (c) creates relational autonomy, and (d) expands the scope of what is possible.

**Conclusion:**

The results of the study suggest that telepresence robots can play a useful role in enhancing the caregiving experience for informal care partners in multifaceted ways. Care partners reported positive benefits of having the robot assist their virtual visits. However, further research is needed to determine the sustainability of robot implementation among diverse geographic regions and care home compositions.

## Introduction

Many family members face conflicting responsibilities and negative consequences while caring for loved ones in long-term care (LTC) homes. In 2018, 25% of the Canadian population (7.8 million Canadians) aged 15 and older informally provided care for families or friends with a long-term mental or physical condition, disability, or age-related needs.^1^ More than half of these individuals providing care were aged 45 to 64, and almost a quarter were 65 and older.^1^ Globally, the International Alliance of Carer Organizations estimates that there are over 64 million informal caregivers worldwide.^2^ In this article, we will adopt the term “care partners” to describe families or friends who provide informal care.

Informal care or caregiving is ongoing, unpaid care and support for another individual (usually family or friend) without payment or formal training. Informal caregiving occurs in both community and LTC settings. A report by Arriagada (2020) indicates that more than half of care partners described their caregiving experiences as rewarding. However, being a care partner can simultaneously become a source of chronic stress and lead to declines in an individual's physical, social, and mental health.^1^ Due to providing informal care, many care partners may not be able to pursue paid work or engage in other activities (e.g. leisure), creating financial burdens as well as consequences for physical, social, and mental well-being. Subsequently, many care partners develop care partner burden and burnout. For instance, care partners assisting with care regarding medical appointments were more likely to face care partner burden than those uninvolved with this responsibility.^3^ In addition, an overwhelming majority of LTC residents have at least one neurological condition, such as dementia: 62.6% of LTC residents were diagnosed with dementia in BC in 2022–2023, with 60.8% of all LTC residents receiving this diagnosis in Canada.^4^ Informal care partners of older adults with dementia report higher mean hours per week of informal care and a higher likelihood of experiencing distress compared to care partners of other older adults.^3^ Negative consequences of care partner burden are especially significant for women, who account for almost two-thirds of all care partners in Canada^1^; Older women caring for spouses spent the most hours on informal care, contributing 20 or more hours of care per week.^1^ This trend is reflected worldwide, as women contribute to around 71% of global informal care hours.^5^ A study by Gagliardi et al. (2022) showed that adult children's care partners were more likely than spouse care partners to perceive negative effects on health and feel unappreciated and trapped with care tasks.^6^ The negative impact of the care burden threatens the health of care partners and their relationship with and ability to support their loved ones.

Due to the rising complexity of needs, many older adults must transition from ageing at home to living in an LTC home to receive an appropriate level of care. While supporting this “forced” transition, care partners of LTC home residents face unique challenges, for example, guilt about placing their loved one into LTC, broken familial commitments with their loved ones, the loss of lifelong company at home, and the disrupted sense of purpose as a caregiver.^7,8^ As care recipients transition to LTC, care partners experience a change in their caregiving roles and the type of care they can contribute to their loved ones.^9,10^ They must surrender a level of control and involvement in day-to-day activities of caregiving to LTC staff, creating frustrations for care partners who do not feel adequately included in care conversations and decision-making^6^ or trust the care “system,” especially after COVID-19.^11^ Some care partners have reported sorrow and self-blame for moving family members to LTC homes.^12,13^ Following the transition into LTC homes, many informal care partners continued to offer physical, emotional, and mental care for their loved ones in LTC homes^14–16^; however, these care partners’ contributions are often ‘invisible’ and unrecognized by the healthcare system.^17^ There may be power imbalances^16^ and mistrust between informal care partners and formal healthcare providers regarding care for residents in LTC homes.^13,18^ Yet, informal care partners often wish to stay involved in the residents’ care, be recognized as a source of knowledge, and feel valued as a key support in sustaining care for their loved ones.^10,19^

During the COVID-19 pandemic, informal care partners of residents living in LTC homes encountered unexpected challenges due to health risks, changing care home protocols, and government policies.^14,20^ Many care partners noted that the pandemic exacerbated stress on the underfunded LTC home workforce, which prompted their desire to assist staff and supplement resident care.^19^ However, recurring social distancing protocols excluded and devalued their work.^19^

In addition, social isolation restrictions and outbreaks in LTC homes disrupted social connections between care partners and residents. As information about their family members’ pain levels and overall well-being became challenging to access, informal care partners experienced worry, stress, and anxiety.^15,20^ An essential need for informal care partners was the ability to remain connected to their loved ones in LTC during the COVID-19 pandemic,^20^ in-person or virtually. Virtual visits between loved ones and care partners can help care partners better understand the resident's condition while maintaining a sense of connection; however, it was challenging for care partners to connect with residents virtually without staff assistance and support.^20^ Due to social distancing restrictions, it was also difficult for care partners to stay connected with residents via face-to-face visits.

**Figure 1.**
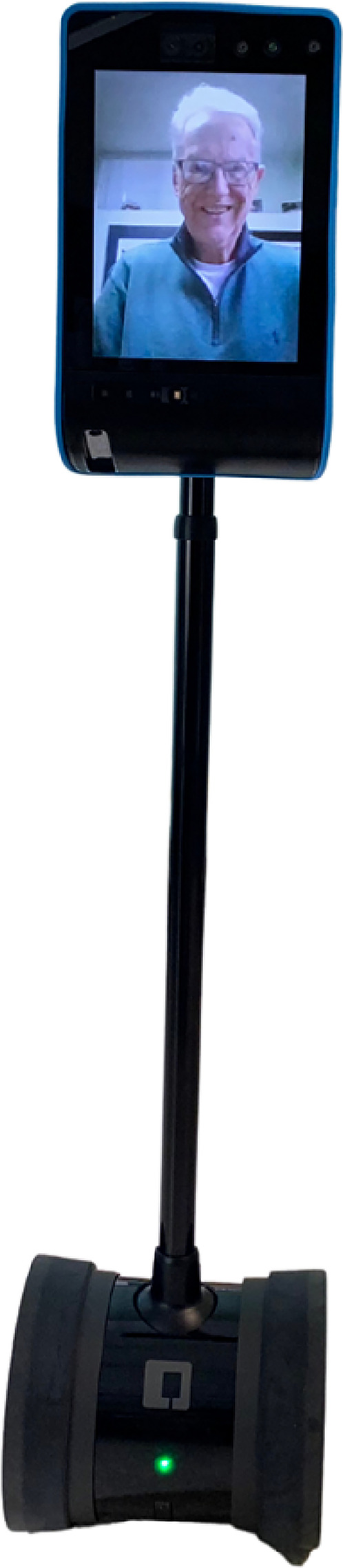
A telepresence robot.

There has been increasing use of technology to virtually connect residents in LTC with care partners, especially during the COVID-19 pandemic. For example, using phone, texting through phone and internet,^21^ and video conferencing communication technology such as handheld tablets and stationary screens.^22,23^ One emerging method to help care partners and LTC residents remain connected is through telepresence robots^24–26^ (see Figure 1). This type of social robot consists of a tablet connected to a pole on wheels. Care partners can meet residents virtually with a stable internet connection anytime and anywhere. Several factors differentiate virtual visits on the telepresence robot from virtual visits over Facetime or Zoom through handheld devices or stationary screens, phone calls and text messages. Telepresence robots allow care partners to control and drive the robot remotely with a smartphone, tablet, or computer. As a result, care partners can move the robot to meet residents where they are, adjusting the robot without placing any burden on the resident. For instance, care partners can modify features such as the height, volume, and proximity of the robot to ensure full visibility of the caller and accessibility to the conversation. Residents do not need to encounter challenges adjusting the camera or navigating different buttons for video calls using tablets.^22^ This adaptability also allows the telepresence robot to create a sense of presence and immersion in the environment, which is generally limited with phone, text messages or tablet calls.^26^ Additionally, telepresence robots enable care partners to call in independently, without needing assistance from staff, thereby enhancing privacy between residents and their families.^26^

Telepresence robots have been explored in a few studies to potentially support family–resident connections.^24,26–28^ However, these studies shared findings on residents’ perspectives of using telepresence robots, including how the robots facilitated social connections between residents and family members. There are limited in-depth explorations on how these robots support informal care partners’ needs and the engagement of informal care partners in resident care at LTC homes.

## Theoretical framework

To explore the experiences of informal care partners using telepresence robots and the ability of these robots to support informal care partners of residents in LTC, we adopted a caregiver-centered care model^29^ in the synthesis and analysis of our findings. “Caregiver-centered care” is defined as “person-centered care*”* for care partners.^30^ The model enables healthcare providers to view the needs of residents and care partners both separately and as a dyad.^29^ Six domains of the healthcare workforce's competencies can be leveraged to support residents’ and care partners’ needs: (a) recognizing the care partners’ roles, (b) communicating with family care partners, (c) partnering with family care partners, (d) fostering resilience in family care partners, (e) navigating health and social systems and accessing resources, and (f) enhancing the culture and context of care.^29^ The caregiver-centered care approach recognizes the value of partnership with care partners and highlights the importance of addressing their needs and preferences.^29,30^ Using this model, we analyzed how telepresence robots could help support and engage informal care partners of residents living in LTC homes during the COVID-19 pandemic.

Our study aims to answer two main research questions. (a) What are the experiences of informal care partners’ using telepresence robots to deliver care to their loved ones living in LTC homes? and (b) How may the robot support informal care partners’ own needs, engagement, and involvement within LTC homes?

## Methods

We conducted our study with participants recruited from five urban LTC homes located in British Columbia. Our research program has previously published findings surrounding telepresence robot use from the perspective of residents and staff within these LTC homes.^24,26^ Here, we present analysis of the perspectives of care partners. We used qualitative methods for our study, including semi-structured interviews, bi-weekly check-ins via email, observation, and field notes, believing these methods could help us achieve our research goals. Qualitative methods offer the advantages of providing detailed descriptions of the complexity of informal caregivers’ experiences, illuminating the processes or stories of how robots may support the well-being of informal care partners, and allowing the research team to interpret these experiences. Collaborative Action Research (CAR) principles were used to guide our approach. Under this paradigm, research is meant to be democratic and have a participative orientation to knowledge creation.^31^ As a collaborative process, this type of research is done with the people in the social context, inviting them in as co-researchers.^31^ The purpose of CAR is to address real-world problems through a cyclical process of co-planning, action, and reflection. This approach empowers participants, incorporates diverse perspectives, and creates actionable knowledge that is context-specific and meaningful to the community involved.

### The telepresence robot in our study and training support for family members

The telepresence robot we used was supplied by Double Robotics (Double Robotics, Inc., software version 3.0). It is manufactured in the United States and used mainly in hybrid classrooms and offices to support flexible or remote schedules. As a self-driving, two-wheeled robot with videoconferencing capacity, it uses 3D sensors to detect and understand its surrounding environment to drive and avoid obstacles safely. The robot uses Mixed Reality to add 3D virtual objects into the video stream, such as waypoints and objects of interest. For example, “click-to-drive” markings are provided and can be employed by care partners to navigate the robot in the environment. Compared to other video conferencing software, the robot's two 13 mega-pixel cameras enable care partners to have an ultra-wide view of the environment; care partners can also raise or lower and turn the robot to focus on objects of interest, as well as adjust audio and visuals without requiring help from the LTC resident or staff. The robot battery has 4 hours of runtime, requiring a 2-hour recharge time thereafter. An online booking system (Fleet Management) was also supplied by Double Robotics, which our research team used to schedule and manage virtual visits for care partners, as well as monitor the charging status on the robot. If the robot was not charging or out of battery, our research team either went to the home in-person to charge the robot or notified and asked LTC staff to assist with placing the robot back into the charging station. Families also notified the staff and our research team if the robot was not charged or working. This was a quick request, so we did not receive any refusal or complaints from staff about disrupting or adding to their workload. To facilitate the request, our research team directed staff step-by-step on how to identify if the robot is in the correct position to charge successfully. After scheduling visits via the online booking system, care partners receive an email invitation, in which they can click the link provided in the email to initiate a virtual visit on their end. Care partners who chose to use the robot at their own convenience created a Double Robotics account to access their assigned robot anytime. Although these care partners had 24/7 access to the robot, they scheduled their calls based on their loved one's routines to minimize possible disruptions to the resident's daily life. To further avoid interruptions to care, staff would place a cloth over the robot's screen while providing care to the resident (i.e. toileting, etc.). This cloth blocked the robot's camera view, signaling to families that their loved one was busy and prompting them to call back at a later time.

Care partners each received an introductory in-person training session, written manual, and supportive resources (i.e. training videos, training summary sheets) prior to robot usage. Additional in-person training was provided as needed to support comfortability and successful usage. During the study, care partners had the option of using the robot 24/7 at their convenience or scheduling recurring visits at a set time.

### Setting and participants

The research study took place from May 2021 until August 2023 at five urban LTC homes located in British Columbia, Canada. Three of the homes are publicly funded, one home is for-profit and privately funded, and one home is non-profit and privately funded. GH, JW, LR, and NA led the interviewing of participants from the abovementioned care sites. Purposive sampling^32^ was undertaken with the assistance of LTC staff members, who identified appropriate residents and care partners for our team to contact via email and recruit into the study; we aimed to capture a diverse population of older adults. Our research team was then responsible for providing further details about the study to the participants either in-person or virtually via Zoom, including discussing research requirements, obtaining informed consent, and arranging robot training sessions and virtual visits. A total of 20 care partners took part in the study. See Table 1 for study participant characteristics. This research is part of a more extensive study that explored the implementation of telepresence robots with LTC residents (care recipients) and staff. Thus, residents and staff also provided informed consent for this study and where necessary, we gained proxy advice on participation from families for residents living with dementia.

**Table 1.** Descriptive characteristics of study participants (*N* = 20).

Characteristics	*N* (%)
Age (years)	
21–30	1 (5)
31–40	2 (10)
41–50	4 (20)
51–60	4 (20)
61–70	6 (30)
71–80	2 (10)
Sex (self-report)	
Male	4 (20)
Female	16 (80)
Ethnicity	
Caucasian	10 (50)
East Asian	10 (50)
Relationship to LTC Resident	
Spouse	1 (5)
Daughter	12 (60)
Granddaughter	1 (5)
Niece	1 (5)
Family friend	1 (5)
Son	3 (15)
Son-in-law	1 (5)

Before the study's commencement, the interviewers did not establish an existing relationship with the participants. Through discussing research requirements and obtaining informed consent, participants developed an understanding of our research aims, including our reasons for interest in the research topic. Trust was built between interviewers and participants after the project commencement, as the former consistently taught the latter to use the robot, performed troubleshooting, addressed the latter's needs, and understood the goals and reasons for the latter to participate in this study. One challenge care partners faced was mastering the robot's self-driving function. Maneuvering the robot with confidence required practice, so we provided follow-up in-person training with families to offer support and practical advice. Other troubleshooting efforts included ensuring the robot was placed in a location with strong Wi-Fi connectivity and convenient access for calls. Each room varied in terms of available Wi-Fi strength and space. Depending on the situation, additional time was spent on setting up the robot and monitoring for potential improvements (i.e. adding a router).

### Research team

The research team included six student researchers, a patient partner, two family partners, and an academic supervisor, totaling 10 people. GH is a female undergraduate student, SK is a female medical student, and JW, LR, and NA are female graduate students. AH is a male undergraduate student. Student authors came from different backgrounds, including public health, medical science, nursing, and engineering. Student authors also received training on conducting qualitative interviews and collecting data under the supervision of LH, a female researcher and academic supervisor. The patient partner is a male older adult with lived experience of dementia. Two female family care partners are informal care partners of older adults living with dementia.

### Data collection

This qualitative study collected data via several methods, including semi-structured interviews, bi-weekly check-ins via email, observation, and field notes. Semi-structured interviews were conducted with care partners and residents individually and together as dyads.^33^ Each interview lasted about 20 to 30 minutes and was carried out in person at the LTC home or via Zoom, depending on the care partners’ preference. Questions in the interview guide were open-ended (see Table 2), seeking to understand what care partners liked or disliked about using the telepresence robots. These questions were developed with the input of family care partners, who collaboratively helped us refine the questions for interviewing. Interviews were audio-recorded with participant consent and were transcribed verbatim by the research team. Bi-weekly check-ins via email were undertaken with care partners to supplement their understanding of their ongoing experiences with using the robot. Field notes taken by interviewers from onsite observations provided additional reflexive thoughts to the data. Semi-structured interviews were also conducted with residents (care recipients) and staff to investigate their experiences. A total of 9 residents and 21 staff members participated in this study. Their experiences have been reported in separate papers.^24,26^

**Table 2.** Interview guide.

**#**	**Questions**
**1**	How do you feel about using the telepresence robot to visit with your loved one?
**2**	Is there anything you like or dislike about it?
**3**	What kinds of challenges or supports have negatively or positively affected your experience?

**Table 3.** Example of thematic analysis**.**

Quotations	Code	Subthemes	Themes
**“Being with her every day is ‘wow, this is great’. Seeing her with your own eyes, all her movements, her expression… without it [the robot] I cannot see her everyday.**	Monitoring loved one's condition	Addressing care partner burden/relief	“I can see how she's really doing”: the robot decreases care partner burden”
**“She, in the care home, most of the time, they are quite alone. They don’t have enough staff to keep the residents busy. With the robot, it really helps. We spend time with the resident, keep them occupied.”**	Contribution to care	Promoted continuity in relationships, inclusiveness, and togetherness between resident and care partner	“I can do more:” the robot expands the scope of what is possible

**Table 4.** Four themes and subthemes.

Themes	Subthemes
“I can see how she's really doing”: Decreases care partner burden	Monitoring LTC resident's condition
	Providing accompaniment, connection and presence
“They let me know”: Facilitates relationships between care partners and staff	Feeling valued by staff
	Building trust with staff
“To not have to impose on people”: Creates relational autonomy	Reducing dependence on staff
	Enhancing control over care family–resident relationship
“I can do more”: Expands the scope of what is possible	Increasing family inclusion and involvement
	Strengthening a sense of purpose

During each family care partner's virtual visit with their loved one, one to two trainees (student researchers from our team) observed their reactions to the robot, assessing their engagement and troubleshooting any issues that arose. Moreover, trainees compiled field notes after the interviews, check-ins, and observations. The aforementioned data collection methods enabled the team to gain rich information and deeper insights into the real-world experiences of family care partners using the robot for virtual visitation and the family–resident interactions in the LTC settings. All participation was voluntary. During the telepresence robot implementation, the care partners of two LTC residents dropped out of the study. One participant expressed that because their loved one was not receptive to the robot, they were not able to benefit from its usage and subsequently withdrew from the study. This participant was excluded from our collected data. Another participant's loved one was later discharged from the unit they lived at and subsequently withdrawn from the study. This participant had already completed data collection with us, so we included them in our collected data. Data collection was completed upon reaching a point of data saturation, meaning there was sufficient data collected to adequately address the research questions.^34^ At this stage, no new information, themes, or insights appeared to emerge from additional data collection, suggesting that the data collected captured a comprehensive picture of the topic being studied. Additional data collection was unlikely to add value to the research, so identifying data saturation improved research efficiency by preventing the collection of excessive data that may not be the best use of time or resources.^32^ Transcripts were not returned to care partners participants for review to avoid placing additional demands on their time.

### Data analysis

We followed the principles of reflexive thematic analysis, which involves six steps.^32^ Step 1: All authors read and re-read the transcripts to familiarize themselves with the data. Step 2: The student author (GH) generated initial codes by using concepts illustrated in the data transcripts (inductive approach) and by consulting salient concepts that exist in the literature (deductive approach). Another student author (JW) reviewed and refined the codes, assigning transcribed text to the relevant codes with GH. Once the codes were finalized, all authors reviewed the codes, including the academic supervisor (LH). See Table 3 for examples of how data was coded. Step 3: Initial themes were generated using the finalized codes and the associated extracted data. Step 4: All authors discussed the data findings and refined the themes accordingly during the large team meetings involving patient partners, family care partners, LTC staff, and the research team. See Table 4 for the finalized themes. Step 5: All authors worked together to finalize the data and select quotations for the final write-up. Step 6: Authors worked in pairs to write the first draft of the manuscript, receiving guidance from the academic supervisor (LH). All authors reviewed the manuscript, making edits and generating several iterations of the manuscript in preparation for manuscript submission.

### Rigor

We took several measures to ensure rigor in our study. To make sure we understood the meaning of our interviewees, we asked clarifying questions, for example, “Let me know if we understand you correctly. Did you mean by…” to double-check with our participants. Also, we made efforts to ensure our patient and family partners were meaningfully engaged and contributed to the study. We consistently practiced team reflexivity throughout the study. This includes creating a safe environment for patient and family partners to contribute their voice. They were invited to all research meetings, and when they attended meetings, they were given sufficient time and opportunities to speak. Patient and family partners were always offered multiple ways to share concerns or thoughts about the study and paper writing, including email, phone call, Zoom, and text message. Our consistent discussion with patient and family partners in data collection and analysis challenged the biases and assumptions of trainees. For example, researchers considered privacy as one of the barriers for staff to implement the robot, when staff were giving personal care to the resident and the family was visiting through the robot. A patient partner challenged researchers, saying that privacy is not a barrier unique to the robot; privacy concerns also arose when family visited residents receiving care in-person. Through team-based discussion, our team developed a mitigating strategy that addressed privacy –by providing staff with a cloth to cover the robot's camera or by placing the robot in a more appropriate corner of the resident's room.

LH, an experienced nursing professor, provided expert guidance in data analysis, ensuring methodological rigor. To enhance transferability, we incorporated diverse perspectives from people of different types of LTCHs, ensuring thorough documentation of the research context, and providing detailed descriptions of our procedures and findings to enable other researchers to apply our insights to similar settings.

### Ethical considerations

The study obtained ethical approval from the local University and Health Authority (H22-00659). All participating care partners (and care recipients) signed written informed consent forms. Pseudonyms were employed to protect their identities. The robot itself cannot record or store any information, ensuring the privacy of participating individuals. Similar to a face-to-face visit, care partners using the robot only have access to the conversation while it is occurring live.

## Results

A total of 20 informal care partners participated in this research study. Women made up 80% of the care partners in our study, including a daughter (*n* = 12), a granddaughter (*n* = 1), a niece (*n* = 1), a family friend (*n* = 1) and a spouse (*n* = 1). The remainder of the care partners consisted of a son (*n* = 3) and a son-in-law (*n* = 1). These care partners supported residents living in five urban LTC homes in British Columbia. Care partner characteristics are presented in Table 1.

Our analysis identified four main themes that capture the experiences of informal care partners using telepresence robots with loved ones residing in LTC settings during the COVID-19 pandemic: (a) decreased care partner burden, (b) facilitated relationships between care partners and staff, (c) created relational autonomy, and (d) expanded the scope of what is possible

### Theme 1: “I can see how she's really doing”: the robot decreased care partner burden

Multiple care partners shared the importance of accurately understanding their LTC resident's daily wellness and condition, especially following the resident's transition into LTC. Moving into LTC meant greater difficulties with monitoring the resident's care and condition. The worries associated with this transition were further heightened by the effects of the COVID-19 pandemic, which became a continuous threat to both health and visitation opportunities available to care partners. Rosie, the daughter of one resident, shared the difficulty of taking care of her young children while trying to make time to visit her father. By increasing the flexibility and convenience of connection, the telepresence robot created the opportunity for Rosie to experience both connection and relief from care partner burden. Rosie shared:

I feel like I’m drowning in many ways as the juggling is quite hard these days with my kids, work, and, of course, my dad being unwell. I am grateful to have access to the robot as it allows consistency in me seeing him… my last visit [through the robot] with my dad was sad for me as he seemed very drugged up. I managed to get his old smile back, which made me happy.

Moments like these illustrated how impactful it was for care partners to continually and visually witness the wellness of their LTC residents – even if they could not observe it in person.

Mina shared a similar experience, highlighting the robot's unique ability to enhance her visits with her mother:

It has been beneficial because we’re able to see her slouching down in her chair and her very poor posture because of that. I could see she had an injury on her leg; she showed me that. Seeing her facial expressions or she looked to [staff] for an answer, and even being able to see what she's looking at outside her room, I found all of that really really helpful… I can see how she's really doing. We have a better conversation with the robot than just with a normal phone call… it's more insightful; you can observe specific behaviours.

Anna, a care partner to her mother living with dementia, corroborated Mina's experience with her own:

We can have peace of mind. Being with her every day is ‘wow, this is great.’ Seeing her with your own eyes, all her movements, her expression.

Rosie's, Mina's, and Anna's experiences captured the robot's ability to provide care partners with a sense of presence and an ongoing grasp on their loved one's level of comfort and quality of life. The reception of these feelings and knowledge was associated with decreased care partner burden; the robot enabled care partners to remain connected to the things that mattered, as family members supporting loved ones living in LTC: understanding their loved one's joys, pains, capacities, and what contributes to these matters.

### Theme 2: “They let me know”: the robot facilitated relationships between care partners and staff

One concern that many care partners face is feeling unseen, undervalued or excluded by staff, especially when it comes to timely communication or shared decision-making surrounding their loved one's care. During the COVID-19 pandemic, the already strained LTC workforce faced heightened staff shortages and turnover rates, creating gaps in care partner–staff communication and contact.^35^ Throughout this difficult period of time, the telepresence robot created new opportunities for staff and care partners to engage with one another. During our conversation with Melanie, she expressed:

I have spoken to some of Dad's care aides while they are tending to him as we use the robot, and all have been wonderful, especially [pseudonym]. And yes, they are letting me know of updates while we are using the robot, but also give us time to speak alone without listening [in].”

Although staff are not required to provide assistance with the robot, sometimes care partners called in via the robot while staff members were caring for the residents. In some instances, staff members provided updates about the resident on the spot or took the chance to ask care partners for their input on a relevant issue. The robot makes care partners more visible in the LTC home, creating opportunities for care partners to stay informed as well as participate in direct care and shared decision-making. Staff asked care partners for their preferences and permission regarding care, establishing a sense of relationship, partnership, respect, and trust. Care partners may develop greater confidence in staff by witnessing the staff's commitment to aligning care with the expertise of care partners, who know the resident best. The telepresence robot can create additional opportunities for care partners to engage with staff as a partner in care and feel valued as a key source of knowledge.

Anna recounted that when she started using the robot, she witnessed the commitment that several staff members felt towards her loved one. She shared:

So far, [pseudonym] has done a really beautiful job. She gets her way to let the staff accept that this [telepresence robot] is good for the residents.

Anna also described instances when the staff actively facilitated virtual visits between her brother and her mother:

Whenever my mum has a companion [staff], and he [my brother] will call in, the companion will encourage my mum, saying, “he is your son!”

The contribution from the staff was impactful, as Anna's mother experienced difficulties with recognizing her son, who was not able to visit her often in-person. Anna explained the sense of solidarity that she built with staff via the robot usage; witnessing the staff's motivation to support her loved ones with robot usage enhanced the sense that they shared a common goal: enhancing the resident's quality of life. Communication and collaboration between staff and care partners promote the trust that safe, personalized, and effective care will continue to take place even when care partners are not there. This is particularly significant for care partners of individuals with dementia, such as Anna, who become the voice and advocate for their loved one's needs. Thus, if staff buy-in is present, the robot can be used as a tool for building trust, facilitating relationships, and improving interpersonal communication - in a way that is responsive to care partner values and wishes, as well as patient needs. Following robot usage, both Melanie and Anna highlighted the support that they received from staff members. These experiences underscore that with the greater frequency and flexibility of virtual visiting (that the robot facilitates access to) also comes the potential for strengthening person and family-centered care via increased opportunities or touchpoints for staff and care partners to communicate and collaborate.

### Theme 3: “To not have to impose on people”: the robot created relational autonomy

During transitions into LTC, care partners are assigned a new role: visitor. Care partners are no longer the primary caregivers; they are expected to trust and depend on care staff to provide care and request support for their LTC residents. However, many care partners report feeling burdened when requesting staff for answers and assistance, often commenting on how busy and overworked the staff are. The COVID-19 pandemic further exacerbated these worries. Yet, care partners felt uninformed or excluded if they did not advocate for these measures. While using the robot, several care partners pointed out that, unlike an iPad or phone, using the robot did not require staff assistance or demand effort from the LTC resident (i.e. holding up a device). Sally noticed this difference in the robot, sharing:

It’s been tough to have to rely on people and to impose on people, to you know [call]. They [the staff] have to be available, and I know they’re busy too, right? So it’s [using the robot] been fun to help them as well, in that they don’t have to be there to facilitate it.

Sally's experience highlighted the robot's ability to facilitate the transition from home to LTC, as via the robot, care partners can maintain a level of involvement that does not require reliance on or permission from staff, simultaneously reducing the potential for delayed action. Using the robot prevented care partners from feeling like a burden to staff and enabled them to advocate for and access what they needed or valued. While using the robot, Anna provided insight into how it felt to not rely on staff for virtual visitation:

You will feel more comfortable when you don’t have a time limit. It's one of the greatest things to happen on earth right now.

The robot usage provided Anna with a reliable way of contacting her mother at suitable times and circumstances without external assistance. It directly countered the visitation restrictions that COVID-19 mandates imposed on care partners. In effect, Anna highlighted that the robot is an enabler of family agency and relational autonomy; it offered a sense of stability and certainty over the time they could spend together, while minimizing disruptions to staff workflow.

### Theme 4: “I can do more”: the robot expanded the scope of what is possible

Care partners often express their desire to continue contributing to the care of their loved ones when they enter LTC. With the pandemic, social distancing mandates made this inclusion difficult. However, while using the robot, several care partners pointed out novel experiences they could share with their loved ones. Marie shared an experience that she had with her father:

So we use the robot to see him work with [his occupational therapist] and see how she trained him [on using his wheelchair]. And so we followed him around on the robot while he was like going around in the hallways and going to his room and then parking it, and just like she got him to do a little obstacle course, and I was just able to follow along with the robot and just see him use it. So that was really cool because if you didn’t have that, you know, like, I wouldn’t have had the chance to do that since we’re not there in person.

The robot's unique ability to be driven by its callers enabled Marie's family to closely follow her father's actions. Marie expressed that this experience not only reassured her that the wheelchair was a good fit but it also meant that they could invest in it without any worries. Marie's experience underscored how the robot provided the chance to more closely involve her family in important care decisions regarding her father. This opportunity for shared decision-making led to a care decision that not only supports the mental health of the care partners but also reduces the risk of wasting financial resources.

Marie also appreciated being able to share parts of her personal life with her father via the robot. She expressed:

My parents know they’ve been here before and know I have a cat. They asked, “Where's the cat?” I even took my iPad and showed the cat to them… so it was good that I could show them more when I was doing the robot with them.

Being able to contribute to and give back pieces of joy to loved ones was a common theme among care partners. Anna shared how she felt about exercising with her mother using the robot:

Let's go for a walk; exercising with her through the robot satisfies me! She is in the care home, and most of the time, they are entirely alone. They don’t have enough staff to keep the residents busy. With the robot, it helps. We spend time with the residents, and keep them occupied.

For Anna, using the robot with her mom provided a sense of purpose and a chance to intervene if or when there was a chance to support her mother. Rosie, who felt guilty about finding it difficult to manage time between her children and her father, was able to soothe her worries by using the robot to bring her family together:

Rosie: “Hi G, go muah to Grandpa. Blow him a kiss! Go muah!”

Rosie: “Oh, she just blew you [George] a kiss!”

Elder granddaughter: “Hi, grandpa!”

George: “Hi, honey!”

Rosie: “Tell Grandpa what we brought him. What did we bring him?”

Granddaughter: “We brought grandpa a cake!”

During this visit, we observed that the robot promoted continuity in relationships, inclusiveness, and togetherness among multiple generations of the family. Based on these experiences, care partners communicated that the robot reassured them of the ability to maintain a meaningful role in their loved one's care. As a tool that facilitated care partner inclusion, the robot helped care partners to contribute more than they otherwise could have, encouraging a greater sense of purpose and certainty that their loved one's needs were being addressed.

## Discussion

Our findings offer insights into how technological innovations can support the underappreciated and often invisible population of informal care partners in LTC. Notably, many of the themes captured are interrelated and mutually reinforcing. The capacity to do more for the LTC residents without requiring help from the staff alleviates the worry that many care partners feel about meeting their loved ones’ needs without overburdening staff. When care partners encounter staff via the robot and experience inclusion and shared decision-making, the care partner–staff relationship can deepen, building trust and mitigating the care partner burden. In this section, we discuss how our findings relate to (a) enhancing the quality of care partner involvement, (b) supporting the recognition of care partner needs, and (c) shifting the culture of care by reframing attitudes towards care partner roles in LTC.

First, the robot promotes the inclusion of additional family members (i.e. intergenerational connections), the flexibility of time accessed (i.e. flexible daily patterns and rhythms), the facilitation of collective activities, and the enhancement of care partner involvement. In LTC homes, this inclusion is essential; care partners who live far from the home face limited opportunities to speak with staff, making it challenging to develop a meaningful understanding of the LTC resident's condition or contribute to care. For LTC residents living with dementia, care partner–staff communication becomes even more critical, as the resident may be unable to express or advocate for their own needs. Usually, care partners can expect to reliably communicate with staff through scheduled care plan meetings, which happen only once every 3 months in one of the homes studied. Suppose care partners can speak with staff outside of these care meetings. In that case, they are most often talking with management staff rather than with direct care workers, that is, nursing assistants, who most closely support and understand their loved one's condition.^36^ As care partners used the robots, we observed that the virtual visits between the LTC resident and care partner introduced opportunities for care partners to interact with staff - and most often with the direct care workers who understand their loved ones the best. The robot created additional touchpoints for these care partners and staff members to engage, enhancing the frequency and depth of input that care partners could contribute and also receive. This finding builds on previous literature, in which family members raised the telepresence robot's potential to put a face to the voices of staff and keep family more involved in their loved one's care.^25^ A study by Niemala et al. corroborates that care workers noticed the difference that telepresence robots made, sharing that additional touchpoints helped them explain issues better and improve their service within LTC settings.^37^ In effect, the robot increases visitation opportunities and acts as a bridge between staff and care partners, increasing care partners’ visibility, voice, and participation in LTC settings. According to Parmar et al., 2020, this bridge effectively strengthens the staff's caregiver-centered care competencies, including understanding the value of care partner contributions, the benefits of including care partners on the care team, and the importance of fostering collaborative relationships with care partners.^29^ The significance of care partner–staff relationships is emphasized by previous research, which illustrates how increased visitation opportunities and flexible visiting hours enhanced collaboration between healthcare providers and family members, which supported family involvement in activities surrounding direct care.^38^ Thus, the robot can act as a tool that increases care partner–staff communication, mutual understanding, and partnership, enhancing a model of caregiver-centered care in LTC settings.^29^

Second, the robot's inclusive nature created novel opportunities to recognize and support care partners’ needs and goals. One of these needs includes relieving the care partner's burden or stress.^39^ The experience of caring for loved ones living in LTC during COVID-19 was particularly psychologically stressful. The robot helped mitigate this stress by enabling care partners to consistently monitor their resident's condition without reliance on staff, facilitating a timely understanding of the resident's changing needs and the ability to respond to these needs in real time. Through enhancing opportunities for care partner inclusion and involvement, robot usage strengthened care partners’ sense of agency and competence in caring for their loved ones. Strengthening this sense of competence supported the well-being of care partners by reducing uncertainty surrounding whether or not their loved ones were receiving the care they needed. This idea builds on previous literature, which describes how increasing a caregiver's competence and confidence in providing safe and effective care to their loved one can indirectly reduce stress via an increased sense of certainty and control.^40^ Existing literature details the link between increased family involvement and enhanced favorable family member experiences and satisfaction, including care partner quality of life.^41–43^

Further, since robot visits emerged organically in the daily life of the LTC resident, care partners were able to witness how care staff integrated into the LTC resident's routines—as opposed to formal care plan meetings, where staff are transferred from real-life settings to a meeting room to discuss the resident with care partners. Care partners could witness the daily routines and rhythms that their loved ones adopted each day and contribute to care with staff rather than hear about it indirectly from staff. This created an additional level of comfort for care partners. Thus, the value of increasing and improving care partner–staff engagement cannot be overstated as a way of supporting the wellbeing of care partners^29^; appreciating the importance of relational care that care partners contribute can better support them not only in their caregiving roles, decisions, advocacy, and care management, but also in their own wellbeing.^29,43^ Care partners face physically and emotionally demanding work associated with caring for their loved ones. These demands heighten in the context of caring for someone living with a serious illness, such as dementia. The robot can enhance the caregiver experience by supporting the staff's caregiver-centered competencies - better enabling the formal healthcare system to recognize, value, and support care partner needs and wishes for care.^29^

Third, the robot can positively reshape the culture of care: the way staff and care partners view the role of informal caregiving for loved ones living in LTC.^29^ The feeling of day-to-day interactions between care partners and staff that the robot can provide, rather than formal care team meetings, can dismantle the power imbalance that care partners often feel within healthcare settings. Baumbusch et al. (2013) describe there is an absence of formal policies that involves the family in decision-making beyond invitations to care conferences, often leaving them feeling powerless.^17^ Thus, disrupting the power imbalance can be influential in reducing barriers to advocacy and building confidence among care partners to engage actively in care. In effect, we witnessed how the robot enhanced the frequency of informal and spontaneous care partner–staff interactions, accompanied by care partner satisfaction and praise for the staff. This finding extends previous literature, which describes technology's ability to make the care process feel more personal.^44^ Further, Gaugler and Mitchell (2021) suggest “informal contacts” or “demonstration of care” that go beyond normal service delivery, that is, care conferences are associated with positive perceptions of and interactions with care staff.^45^ For example, when structured modes of information sharing accompanied bedside handover provided by nurses, care partners were found to better develop trusting relationships with their nurses.^46^ Shared decision-making has also been shown to optimize transitions and family satisfaction in LTC settings.^47,48^ Thus, creating a culture and context of healthcare that reflects the value of care partners is essential to improving care partner–staff communication and collaboration, which can lead to downstream positive outcomes of care.

Lastly, care partners themselves can also be older adults, as in the case of spousal care partners. Ageist attitudes in healthcare settings have been well-documented, placing older adult care partners at risk of negative interactions and outcomes. In fact, ageism among formal care providers was found to be associated with poorer professional and personal contact with older adults.^49^ Despite the fact that much literature explores the impacts of ageism on individuals living in LTC,^50–52^ there is a lack of attention directed towards the vulnerabilities that older adult care partners may face. One understudied area of concern surrounds whether or not ageism mediates the level of inclusion or exclusion that care partners may experience when seeking involvement in care. While ageism and power imbalances between health care professionals and care partners may present an issue in LTC, the robot creates an opportunity to mitigate these concerns. Intentionally enhancing the sense of collaboration between staff and care partners is significant; the robot can help to dismantle the hierarchical provider-patient relationship by facilitating information-sharing that values and empowers care partners as the experts of their loved one's condition. These are key competencies to creating caregiver-centered care, shifting staff attitudes towards valuing the importance of care partners and establishing the practice of sustaining them as partners in care.

Our findings address our research questions with rich data, contributing to the literature in the field by providing new insight into the benefits that telepresence robots can provide to informal care partners. First, care partners who used the telepresence robot to deliver care to loved ones living in LTC experienced collaborative engagement with care staff, enhancing their ability to receive timely updates and contribute to care decisions. Care partners also shared that the relational autonomy provided by the robot enabled them to spend more time with loved ones, deepening their relationships and expanding the scope of possible activities that could be shared together. Second, by enabling care partners to remain more involved in monitoring and delivering care, care partners expressed a greater sense of agency and competence in caring for their loved ones; this sense of agency had huge implications for supporting care partner wellbeing and reducing care partner burden. Telepresence robots present an opportunity to expand the ways in which we think about informal caregiving, creating a medium through which we can uplift the needs and preferences of care partners caring for loved ones living in LTC homes.^29^ Previous literature has primarily focused on the experiences of LTC residents who used these robots. In our study, we elevate a caregiver-centered approach to informal caregiving, challenging the existing ideas of support, involvement, and boundaries that care partners may receive, practice, and face in LTC settings. Although our findings support the robot's use as a supportive tool, it is important to understand that it does not replace human care; it supplements in-person visiting, filling in gaps where needed.

Despite the promising use of telepresence robots, several challenges create barriers to successful robot implementation. The organizational culture of the LTC home can determine whether or not care partners reap the full benefits of robot usage. Without staff buy-in and acceptance of the robot, care partners may not be able to experience the benefits of increased visitation that enhance care partner–staff bonds. Comprehensive structural support, such as training and technical support, and acknowledgement and promotion of successful robot usage, should be offered to increase staff engagement and successful robot implementation.^24^ In addition, each family, which consists of care partner(s) and LTC residents, is unique. Considering family dynamics before deciding who to include in robot usage in care homes is important. The needs of each care partner are heterogeneous, which underscores that technology implementation will not be uniform; it is important to acknowledge the feelings and capacities of each individual care partner and find ways to support them in their desired role(s).

The financing of technology use in LTC may also present a potential challenge. According to Freedman et al. (2005), limited provider resources for technologies that require larger initial investments, and limited reimbursement from insurance companies for the purchasing of new technologies are barriers to financing technology in LTC.^53^ Providers also point out the hidden costs of purchasing technology, such as costs associated with training, upgrading, or motivating staff to adopt new behaviors to support the technology. A lack of discussion about the potential cost-savings that technology, such as telepresence robots, could offer also presents a barrier to investing in technology implementation.^53,54^ The cost-effectiveness and sustainability of long-term technology use, digital equity, and support beyond the completion of a research study, such as ours, needs to be studied further. Analyses about the costs and benefits of technologies can help inform decision-making about investments and provide guidance to insurers interested in covering the costs of such technologies. It may be meaningful to consider including families and older adults in decision-making surrounding LTC investment, that is for technology usage.

### Future research

Although using the robot to support care partners’ involvement in care planning and shared decision-making is promising, it is necessary to point out that many LTC homes do not have the infrastructure in place to support such technology usage. Notably, rural LTC homes face issues with broadband connectivity, which exacerbates the digital divide: the gap in access to modern information and communications technology that can exist between demographics and geographic regions. Future research should further explore the gaps in infrastructure that LTC homes face in order to facilitate successful telepresence robot implementation. Despite the fact that many questions about its usage remain unanswered, the robot has demonstrated its capacity to facilitate a caregiver-centered approach to care in LTC settings.

### Strengths and limitations

One strength of our study is the diversity of care partner ages that are included within our participant sample. Our study also integrates the insights of patient and family partners within the research planning, data collection, joint data analysis, data interpretation, and manuscript writing processes. Our patient partners provide diverse lived experiences about informal caregiving in LTC settings. Close partnership with and participation of study participants was also key, as we used each care partner's input to drive continuous quality improvement. One limitation of our study is that the care partners who were recruited into our study consisted of families who were already interested in robot usage, creating selection bias. In addition, our study's budget and timeline restricted our knowledge to a limited time frame. We could not explore what scaling up robot use would look like or understand the sustainability of long-term robot usage that functions independently of a research team's support. Another limitation is that this paper does not explore the staff perceived level of work associated with the telepresence robot. The experiences of staff from this study are explored in a separate paper.^24^

## Conclusion

This study used qualitative methods and a caregiver-centered model to analyze the experiences of care partners using telepresence robots to support loved ones during the COVID-19 pandemic. Care partners viewed the robots positively, experiencing the opportunity to facilitate care partner burden relief, strengthen care partner–staff relationships, enable relational autonomy, and restore and elevate the scope of what is possible. Telepresence robots have the potential to enhance the caregiving experience and better support care partners at any stage of the care journey.
